# Reduction in Seizure Generalization Associated With Long‐Term Low‐Dose Immunomodulatory Therapy Using Prednisolone and Methotrexate in Rett Syndrome: A Case Report

**DOI:** 10.1155/crpe/7163898

**Published:** 2026-06-12

**Authors:** Naylya Djumaeva

**Affiliations:** ^1^ Outpatient Department, Research Institute of Virology of the Republican Specialized Scientific and Practical Medical Center of Epidemiology, Microbiology, Infectious and Parasitic Diseases, Tashkent, Tashkent State, Uzbekistan

**Keywords:** epilepsy, generalized seizures, methotrexate, mutation, neuropsychiatric disorder, prednisolone, Rett syndrome

## Abstract

**Objective:**

Rett syndrome is a severe neurodevelopmental disorder most commonly associated with pathogenic variants of the *MECP2* gene and frequently accompanied by epilepsy. Seizures occur in a substantial proportion of patients and may be resistant to conventional antiepileptic therapy. Emerging evidence suggests that neuroinflammatory processes may contribute to seizure propagation and represent a potential therapeutic target.

**Case Report:**

A girl born at term with initially normal early development is described. At approximately 2 years of age, she developed regression of speech and stereotypic hand movements, accompanied by seizures and other neurological features consistent with Rett syndrome. The patient received long‐term therapy consisting of low‐dose prednisolone and methotrexate in addition to anticonvulsant treatment. During follow‐up, a reduction in seizure generalization and a shift in seizure pattern were observed, with generalized seizures becoming less frequent and replaced by brief focal seizures without secondary generalization.

**Conclusion:**

In this single case, long‐term low‐dose prednisolone and methotrexate therapy was associated with a reduction in seizure generalization. Although a causal relationship cannot be established, this observation supports the hypothesis that immunomodulatory mechanisms may influence seizure propagation in pharmacoresistant epilepsy.

## 1. Introduction

Rett syndrome is a severe neurodevelopmental disorder most commonly associated with pathogenic variants of the *MECP2* gene and characterized by progressive neurological impairment following a period of apparently normal early development. However, emerging evidence suggests that this period may also include subtle abnormalities [1–3]. Epilepsy is a frequent and clinically significant feature, often presenting with heterogeneous seizure types and a fluctuating clinical course [4, 5]. Seizure control remains challenging despite the use of conventional antiepileptic drugs. Seizure management in this disorder is further complicated by congenital autonomic and cardiorespiratory dysfunction, which can affect both seizure dynamics and overall clinical stability [6]. In addition, refractory epileptic syndromes, including Lennox–Gastaut syndrome, have also been reported in association with Rett syndrome, further illustrating the heterogeneity and potential severity of epileptic manifestations in this population [7]. In some patients, refractory epilepsy may require alternative therapeutic strategies, including dietary approaches and neuromodulation; however, access to these options may vary across healthcare settings. Increasing attention has been directed toward the role of neuroinflammatory processes in epileptogenesis [8]. Recent advances in the treatment of Rett syndrome, including the approval of trofinetide in the United States and Canada but not in the European Union or the United Kingdom, may support the emerging concept that modulating neuroinflammation and network processes represent a relevant therapeutic approach [9].

Here, we report a longitudinal clinical observation of a child with Rett syndrome and pharmacoresistant epilepsy in whom long‐term follow‐up was associated with a marked reduction in seizure generalization and a transformation of seizure pattern, despite persistence of focal epileptiform activity on EEG.

## 2. Case Presentation

In 2020, the parents of a 3.5‐year‐old child with a clinical diagnosis of Rett syndrome presented her to our clinic. The diagnosis of Rett syndrome was established at the age of 3 years by pediatric neurologists at the Department of Neurology, Institute of Postgraduate Medical Education, Republic of Uzbekistan, based on clinical features consistent with the revised diagnostic criteria proposed by Neul et al., including regression of acquired skills, loss of purposeful hand use, stereotypic hand movements, and the development of seizures [3]. Diagnostics for the presence of the pathogenic *MECP2* variant were not performed due to the lack of the test in our country. At the time of presentation, the child had severe, poorly controlled epilepsy despite antiepileptic therapy with carbamazepine. According to the parents, generalized seizures developed approximately 6 months earlier following an infectious illness and occurred several times a day, including nocturnal episodes. The severity and frequency of the seizures led to multiple hospitalizations. No episodes of status epilepticus were noted.

According to parental reports, early development during the first year of life was largely unremarkable. At approximately 12 months of age, the child demonstrated age‐appropriate vocalizations, including babbling and imitation of animal sounds. At around 18 months of age, the parents noticed the onset of head tremor. At that time, the child was evaluated by physicians and was diagnosed with attention deficit hyperactivity disorder. By the age of 3 years, developmental concerns increased, and the possibility of Rett syndrome was raised for the first time, although the diagnosis remained uncertain at that stage. Subsequently, progressive neurological symptoms became more evident, including behavioral disturbances and stereotypic movements of the hands, which led to a clinical diagnosis of Rett syndrome.

At the time of the first presentation, the child’s clinical condition was severe and included frequent generalized seizures, pronounced stereotypic hand movements, apnea, behavioral disturbances with episodes of aggression and spontaneous screaming, sleep disturbances in the form of episodes of prolonged insomnia, and signs of right‐sided pyramidal insufficiency.

Prior to the described intervention, the patient received antiepileptic therapy including carbamazepine, later replaced (2022) by sodium valproate (Depakine, 300 mg twice daily). Other treatment options, including the ketogenic diet and vagus nerve stimulation, were not implemented, as these approaches were not available within the local healthcare setting at the time of clinical management.

Due to the lack of a satisfactory response to standard antiepileptic therapy and taking into account the child’s general condition, treatment with low doses of prednisolone was started in March 2020 in accordance with an individual low‐dose treatment protocol. After initiation of therapy, gradual clinical improvement was observed. The frequency of seizures decreased, behavioral disturbances diminished, and stereotypical hand movements markedly decreased. The therapeutic effect usually lasted for about 6 months, with exacerbations occurring primarily with concomitant infections, which forced the child’s parents to return to our clinic to initiate the proposed therapy.

In September 2021, low‐dose methotrexate was introduced as an adjunct immunomodulatory therapy. Video‐EEG monitoring, performed approximately 3 months later, revealed focal epileptiform activity originating from the frontal lobes, with no clinical seizures recorded during monitoring. In the following years, the patient continued to be monitored in the clinic, and low‐dose prednisolone and methotrexate therapy were administered periodically, depending on clinical exacerbations. Generalized seizures became significantly less frequent, typically triggered by infections. By December 2025, a marked clinical improvement was observed. Approximately 1 month after resumption of prednisolone and methotrexate therapy, the previously frequent generalized seizures disappeared. Seizures transformed into short focal paroxysms without generalization, occasionally accompanied by hypersalivation. At the same time, stereotypic hand movements resolved, sleep normalized, and behavioral disturbances markedly decreased. The girl became more socially active, willingly playing with her younger sister. At the time of the last examination, the patient experienced only rare generalized seizures, occurring approximately once every 2–3 days, usually triggered by concomitant diseases. Current therapy includes low‐dose prednisolone (5 mg/day), methotrexate (1.25 mg/week), and sodium valproate with therapeutic drug monitoring. During follow‐up, the sodium valproate dose was gradually reduced based on clinical response and serum levels and has been 450 mg/day (divided doses) since December 2025. Notably, seizure control continued to improve during this period, with a significant reduction in seizure generalization despite the dose reduction.

Prednisolone was administered at low doses ranging from 7.5 mg/day to 2.5 mg/day over the period from 2020 to the present. Methotrexate was administered at low doses ranging from 2.5 mg/week to 1.25 mg/week during the same period. Treatment was delivered as intermittent courses, typically initiated approximately once per year during periods of clinical worsening. Each course was followed by treatment discontinuation until the next exacerbation. Dose titration within each course was guided by seizure frequency, clinical status, and routine safety monitoring of laboratory results. The administered doses were lower than those typically used in conventional anti‐inflammatory or immunosuppressive regimens.

### 2.1. Physical and Neurological Examination

At the most recent clinical evaluation, the patient was an 8‐year‐old girl weighing 24 kg and standing 122 cm tall.

During the examination, the child appeared calm and demonstrated preserved visual fixation. She responded to verbal address by turning her head toward the examiner. Spontaneous movements of the upper limbs were present. Hand stereotypies were not observed during the examination; however, according to parental reports, they occur intermittently.

Muscle tone was within normal limits. Tendon reflexes were moderately increased, slightly more pronounced on the right side. Pathological reflexes were not detected. Coordination testing was limited due to difficulties in performing directed tasks.

Cardiorespiratory parameters were stable. Respiratory rate was 22 breaths per minute, and heart rate was 64 beats per minute.

During the examination, a brief paroxysmal episode was observed. The event lasted several seconds and was characterized by a sudden interruption of ongoing activity with gaze deviation and hypersalivation. Frequent blinking was also noted. No change in muscle tone was observed during the episode. Postictal symptoms were not evident.

Video recordings provided by the parents showed similar short paroxysmal episodes during everyday activities, including meals. In one such episode, the child suddenly stopped activity and food dropped from the mouth, accompanied by hypersalivation and blinking.

According to parental reports, generalized seizures that had previously occurred several times daily are now rare and typically appear only in association with intercurrent illness.

### 2.2. Diagnostic Assessment

#### 2.2.1. Electroencephalography

Electroencephalographic studies were performed at various stages of the disease.

The first EEG examination demonstrated abnormalities in bioelectrical activity, with focal epileptiform discharges predominantly in the fronto‐central and frontotemporal regions. The background rhythm was partially preserved but showed signs of slowing.

A follow‐up video‐EEG monitoring performed in December 2021, approximately three months after initiation of methotrexate therapy, revealed diffuse slowing of background activity with dominant frequencies of approximately 6‐7 Hz. Focal epileptiform activity persisted in the frontal regions and was represented by spike‐wave and polyspike‐wave complexes. No clinical seizures were recorded during the monitoring period.

Subsequent EEG examinations performed during follow‐up, including a study in 2025, continued to demonstrate focal epileptiform activity without clear evidence of generalized epileptiform discharges. Despite the persistence of focal epileptogenic activity, the clinical frequency of generalized seizures significantly decreased during the observation period.

Overall, the EEG findings suggested persistence of focal epileptogenic activity with partial stabilization of background rhythm organization over time. Notably, the reduction of generalized seizures occurred despite persistence of focal epileptiform activity on EEG.

Electroencephalographic data and reports are available in the supplementary dataset deposited in the Zenodo repository (https://zenodo.org/records/19736794; DOI 10.5281/zenodo.19050117).

#### 2.2.2. Laboratory Investigations

Routine hematological and biochemical monitoring was performed during the course of treatment.

Complete blood count parameters remained within normal limits (reference ranges in parentheses):

 hemoglobin—128 g/L (110–140 g/L)
 erythrocytes—4.39 × 10^12^/L (3.8–5.2 × 10^12^/L)
 platelets—267 × 10^9^/L (150–400 × 10^9^/L)
 leukocytes—6.89 × 10^9^/L (4.5–13.5 × 10^9^/L).

Biochemical parameters also remained stable. Liver enzymes showed only minimal elevation without clinical significance:

 ALT—38 U/L (10–40 U/L)
 AST—40 U/L (10–40 U/L).

Serum bilirubin levels were within normal limits (5–21 μmol/L).

Electrolyte levels were normal:

 sodium—139 mmol/L (135–145 mmol/L)
 potassium—4.44 mmol/L (3.5–5.0 mmol/L).

Therapeutic drug monitoring demonstrated serum valproate levels in the upper therapeutic range (up to 117 μg/mL; reference therapeutic range 50–100 μg/mL), which guided subsequent dose adjustment during follow‐up.

Although specific biomarkers of infection risk (such as C‐reactive protein or procalcitonin) were not routinely assessed, regular clinical evaluation and standard laboratory monitoring, including complete blood count, did not reveal signs of immunosuppression or increased susceptibility to infection. No clinically significant infectious events were recorded during follow‐up.

### 2.3. Bone Mineral Density

Bone mineral density was assessed by dual‐energy X‐ray absorptiometry (DXA). Lumbar spine measurements demonstrated bone mineral density within the normal range for age, with a *Z*‐score of +1.7. Measurements of the right femur were also within age‐appropriate limits (*Z*‐score approximately −0.3).

A mild reduction in bone mineral density was observed in the left femur, with a *Z*‐score of −1.1; however, this did not reach the threshold for osteopenia. Overall, during the specified observation period, the patient’s bone mineral density remained within age‐appropriate limits, with no signs of clinically significant decrease. Long‐term therapy with low‐dose prednisolone and methotrexate was well tolerated, with no signs of hematological toxicity or clinically significant liver dysfunction.

### 2.4. Therapeutic Intervention

The choice of therapeutic approach in this case was guided by several considerations. The patient demonstrated pharmacoresistant epilepsy with insufficient response to conventional antiepileptic therapy, while alternative treatment options such as the ketogenic diet and vagus nerve stimulation were not available in the local healthcare setting. In addition, emerging evidence suggests a potential role of neuroinflammatory mechanisms in epileptogenesis, providing a rationale for considering immunomodulatory strategies in selected cases.

Based on these factors, low‐dose prednisolone was initiated in March 2020 as an individualized therapeutic approach. In September 2021, low‐dose methotrexate was introduced as an adjunct immunomodulatory therapy for recurrent clinical exacerbations. The treatment strategy consisted of intermittent low‐dose courses with careful clinical and laboratory monitoring, aiming to balance potential therapeutic benefits against safety concerns.

During follow‐up, this approach was associated with a gradual reduction in seizure frequency and improvement in behavioral symptoms. The patient continued to receive low‐dose prednisolone and methotrexate intermittently, depending on clinical relapses. By December 2025, a marked clinical improvement was observed, with the disappearance of previously frequent generalized seizures following the resumption of combined therapy.

### 2.5. Follow‐Up and Outcomes

At the most recent follow‐up, the patient’s clinical condition had significantly improved. Generalized seizures, which had previously occurred several times daily, became rare and were observed approximately once every 2–3 days, usually in association with intercurrent illness.

In addition, a change in seizure pattern was observed. Previously frequent generalized seizures were replaced by short focal seizures without secondary generalization. These episodes lasted several seconds and were occasionally accompanied by hypersalivation and blinking.

Behavioral disturbances markedly decreased. Sleep patterns improved, and stereotypic hand movements became much less pronounced. An improvement in communication‐related behaviors was also noted. The patient demonstrated increased responsiveness to verbal stimuli, improved eye contact, and greater engagement in social interaction. According to parental reports, the child became more communicative and attentive to her environment. These observations are supported by video recordings available in the supplementary dataset.

At the last examination, the patient was receiving the following therapy: prednisolone 5 mg/day, methotrexate 1.25 mg/week, and low‐dosesodium valproate, with gradual dose reduction over time under therapeutic monitoring. No clinically significant adverse effects of therapy were observed.

**FIGURE 1:**
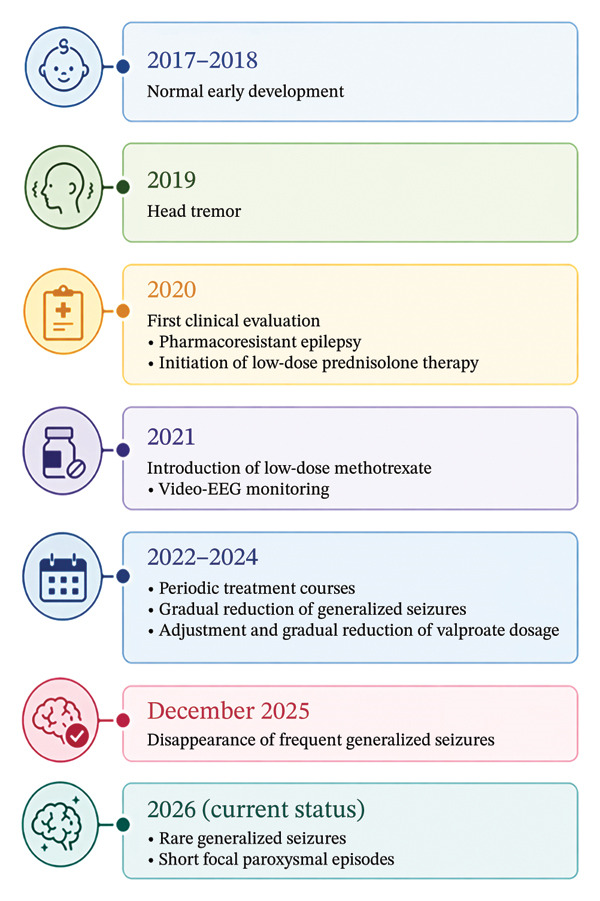
The evolution of the disease course and therapeutic interventions. Clinical timeline illustrating the evolution of neurological symptoms, therapeutic interventions, and changes in seizure pattern during long‐term follow‐up of a child with Rett syndrome.

The evolution of the disease course and therapeutic interventions is summarized in Figure 1.

**FIGURE 2:**
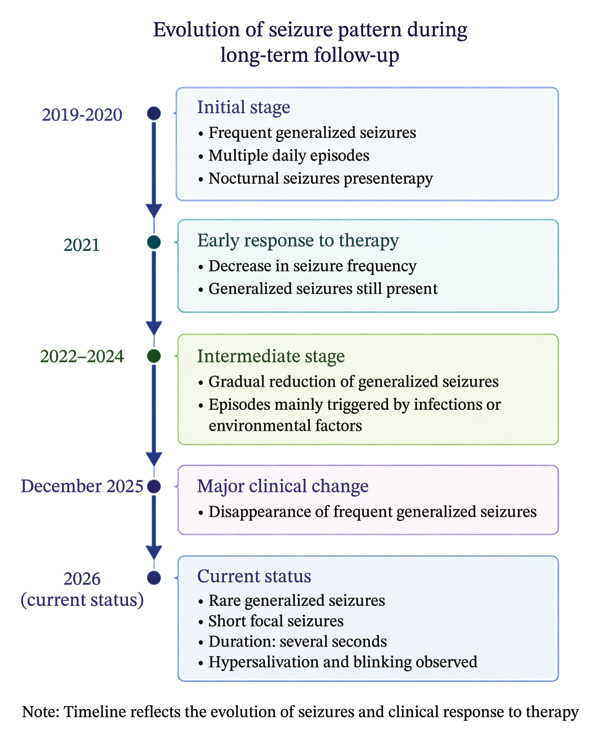
Evolution of seizure pattern during long‐term observation showing transformation of frequent generalized seizures into short focal paroxysmal episodes.

The evolution of seizure pattern during long‐term follow‐up is shown in Figure 2.

## 3. Discussion

Epilepsy is a common and clinically significant manifestation of Rett syndrome, affecting a substantial proportion of patients during the course of the disease [10]. Seizures may occur at different stages and often present with heterogeneous semiology, including both generalized and focal events [11, 12]. In many cases, seizure control remains challenging despite treatment with conventional antiepileptic medications [13]. In the present case, the patient initially exhibited frequent generalized seizures that were poorly controlled with standard anticonvulsant therapy. During long‐term follow‐up, a gradual shift in the seizure pattern was observed in the context of treatment with low‐dose prednisolone and methotrexate administered alongside antiepileptic medication. Generalized seizures became less frequent and were replaced by brief focal paroxysmal episodes without secondary generalization. Importantly, the observed clinical improvement occurred in the context of a gradual reduction in the sodium valproate dosage guided by therapeutic drug monitoring. This observation suggests that the reduction in seizure generalization cannot be explained solely by intensification of conventional antiepileptic therapy and may reflect additional mechanisms influencing seizure propagation.

Notably, electroencephalographic findings continued to demonstrate focal epileptiform activity throughout the observation period. Thus, the reduction in clinical seizure generalization occurred despite the persistence of focal epileptogenic activity on EEG. This observation may suggest a potential effect on mechanisms involved in seizure propagation rather than on the primary epileptogenic focus itself.

Increasing attention has been directed toward the role of neuroinflammatory processes in epileptogenesis. Experimental and clinical data indicate that inflammatory pathways may contribute to neuronal hyperexcitability and facilitate the propagation of epileptic activity within cortical networks [14]. Modulation of inflammatory signaling has therefore been proposed as a potential therapeutic target in selected forms of epilepsy [15–17].

The potential relevance of these mechanisms in Rett syndrome is supported by recent pharmacological developments. Trofinetide, a synthetic analog of the glycine–proline–glutamate (GPE) fragment of IGF‐1, has been shown to modulate synaptic function and neuronal–glial interactions and may reduce neuroinflammatory processes [9]. It should be clarified that trofinetide is approved for use in the United States and Canada; however, it is not currently approved in the European Union or the United Kingdom. Although its exact mechanism of action remains incompletely understood, these findings support the concept that modulation of inflammatory and network‐level processes may influence clinical manifestations in Rett syndrome.

In this context, the use of prednisolone and methotrexate in the present case may be viewed as targeting immune and inflammatory pathways. Both agents possess well‐established immunomodulatory and anti‐inflammatory properties, although their mechanisms of action differ. It is therefore plausible that modulation of inflammatory processes may have contributed to the observed reduction in seizure generalization and improvement in behavioral and motor features.

However, this interpretation remains speculative and cannot establish a causal relationship based on a single clinical observation. Further studies are required to clarify the role of inflammatory modulation in Rett syndrome and related phenotypes.

While prednisolone and methotrexate differ fundamentally in their pharmacological profiles from trofinetide, both agents possess well‐established immunomodulatory and anti‐inflammatory properties. In this context, the clinical improvement observed in the present case, including reduction in seizure generalization and partial improvement in pyramidal signs, may be cautiously interpreted as potentially consistent with modulation of neuroinflammatory pathways. However, such an interpretation remains hypothetical and cannot establish causality.

An improvement in communication abilities was also noted during follow‐up. Although a formal standardized assessment was not performed, the patient demonstrated increased responsiveness to verbal stimuli, improved eye contact, and greater engagement in social interaction. According to parental reports, the child became more communicative and attentive to her environment. These observations are supported by video recordings available in the supplementary dataset.

An important limitation of this report relates to the use of prednisolone and methotrexate in a patient population potentially vulnerable to infections. These agents are not conventionally used for seizure control in Rett syndrome and are generally associated with immunosuppressive effects, which may increase the risk of infectious complications, particularly in pediatric patients with chronic neurological disorders [18]. In the present case, both agents were administered in low doses with regular clinical and laboratory monitoring, and no clinically significant infectious complications were observed during the follow‐up period. Nevertheless, the absence of adverse events in a single patient does not exclude potential risks, and these findings should be interpreted with caution. These considerations underscore that such an approach should be interpreted strictly as an observational finding rather than a recommendation for routine clinical use.

Both prednisolone and methotrexate possess immunomodulatory and anti‐inflammatory properties [19–21]. Corticosteroids have been used in certain neurological conditions associated with immune dysregulation, including specific epileptic syndromes [22]. Methotrexate, when administered at low doses, exerts immunomodulatory effects and is widely used in chronic inflammatory and autoimmune diseases [23]. Although these agents are not routinely employed in epilepsy associated with Rett syndrome, their potential influence on neuroinflammatory pathways may hypothetically contribute to modifications in seizure dynamics. The therapeutic approach in this case was determined by the combination of pharmacoresistant epilepsy, limited availability of alternative treatments, and the emerging concept of neuroinflammatory involvement in epilepsy.

An additional relevant observation in this case was the preservation of bone mineral density despite long‐term administration of low‐dose prednisolone and methotrexate. Corticosteroid therapy is commonly associated with a risk of reduced bone density, particularly in pediatric patients with chronic neurological disorders, including Rett syndrome [24, 25]. However, in the present case, bone mineral density remained within age‐appropriate ranges, with only a mild, nonclinically significant asymmetry observed in the femoral measurements. These findings may further support the tolerability of the low‐dose regimen used in this patient, although conclusions are limited by the single‐case design.

It is important to note that prednisolone and methotrexate are not standard treatments for seizure control in Rett syndrome. Their use in this case represents an individualized therapeutic approach in the context of drug‐resistant epilepsy. Potential risks, including increased susceptibility to infections and neuropsychiatric side effects such as agitation or behavioral changes, must be carefully considered. In the present case, no worsening of behavioral symptoms was observed during treatment. On the contrary, behavioral disturbances decreased, with periods of calmness and improved social interaction reported by the parents and observed during clinical follow‐up. The patient became more socially engaged and demonstrated increased responsiveness to the environment.

This report describes a single clinical observation and does not allow for the establishment of a causal relationship between immunomodulatory therapy and the observed clinical changes. In addition, the natural variability of seizure frequency in Rett syndrome and the influence of concomitant antiepileptic treatment must be taken into account. According to the parents, therapy with low doses of prednisolone and methotrexate, carried out in courses during the specified period, had a positive effect on the child’s condition in terms of the general condition, the reduction of the child’s generalized seizures, the improvement of her social status, and the improvement of her cognitive functions.

Nevertheless, the observed shift in seizure pattern during prolonged follow‐up suggests that further investigation of immunomodulatory strategies in selected cases of pharmacoresistant epilepsy associated with Rett syndrome may be warranted.

A further limitation is the absence of genetic confirmation of the *MECP2* mutation. Although the diagnosis was based on established clinical criteria, the possibility of a Rett‐like phenotype cannot be excluded.

## 4. Conclusion

This case describes the long‐term observation of a child with clinically diagnosed Rett syndrome and pharmacoresistant epilepsy. Treatment with low‐dose prednisolone and methotrexate was associated with a reduction in seizure generalization and a shift in seizure pattern, accompanied by improvements in behavioral and social functioning.

Treatment strategies for epilepsy in Rett syndrome are highly individualized and depend on clinical presentation, response to therapy, and available treatment options. The approach described in this case was applied in the context of pharmacoresistant epilepsy and limited access to alternative interventions such as the ketogenic diet or vagus nerve stimulation.

However, the potential risks associated with immunomodulatory therapy, including increased susceptibility to infections, must be carefully considered. The absence of adverse effects in a single case does not establish safety.

This report has several important limitations, including a single‐case design, a lack of standardized assessment of certain clinical outcomes, and the absence of genetic confirmation of a pathogenic *MECP2* variant. Therefore, the possibility of a Rett‐like phenotype cannot be excluded.

Further studies are required to clarify the potential role of immunomodulatory approaches in selected cases of pharmacoresistant epilepsy associated with Rett or Rett‐like syndromes.

## Funding

The study was conducted without external funding or grants.

## Ethics Statement

Written informed consent for the publication of clinical data, laboratory results, and video materials was obtained from the patient’s parents. All clinical information was anonymized prior to publication. Ethical approval was not required for this case report under local regulations.

## Conflicts of Interest

The author declares no conflicts of interest.

## Data Availability

The data that support the findings of this study are openly available in “Zenodo” at https://zenodo.org/records/19736794; DOI 10.5281/zenodo.19050117. Video recordings illustrating behavioral and communication changes are available in the supplementary dataset (Zenodo repository).

## References

[1] Singh J. and Santosh P. , Molecular Insights Into Neurological Regression With a Focus on Rett syndrome: a Narrative Review, International Journal of Molecular Sciences. (2025) 26, no. 11, 10.3390/ijms26115361. PMC12154281 40508170

[2] Einspieler C. and Marschik P. B. , Regression in Rett syndrome: Developmental Pathways to Its Onset, Neuroscience & Biobehavioral Reviews. (2019) 98, 320–332, 10.1016/j.neubiorev.2019.01.028. 30832924

[3] Neul J. L. , Kaufmann W. E. , Glaze D. G. et al., Rett syndrome: Revised Diagnostic Criteria and Nomenclature, Annals of Neurology. (2010) 68, no. 6, 944–950, 10.1002/ana.22124. 21154482 PMC3058521

[4] Tarquinio D. C. , Hou W. , Berg A. et al., Longitudinal Course of Epilepsy in Rett syndrome and Related Disorders, Brain. (2017) 140, no. 2, 306–318, 10.1093/brain/aww302. 28007990 PMC5278305

[5] Glaze D. G. , Percy A. K. , Skinner S. et al., Epilepsy and the Natural History of Rett syndrome, Neurology. (2010) 74, no. 11, 909–912, 10.1212/WNL.0b013e3181d6b852. 20231667 PMC2836870

[6] Cordani R. , Tobaldini E. , Rodrigues G. D. et al., Cardiac Autonomic Control in Rett syndrome: Insights from Heart Rate Variability Analysis, Frontiers in Neuroscience. (2023) 17, 10.3389/fnins.2023.1048278. PMC10067665 37021139

[7] Olmos García de Alba G. , Gamboa Marrufo J. D. , Rengifo Ramos O. et al., Rett′s Syndrome With Lennox-Gastaut Pattern, Clinical Electroencephalography. (1987) 18, no. 4, 187–190. 3117441

[8] Vezzani A. , Friedman A. , and Dingledine R. J. , The Role of Inflammation in Epileptogenesis, Neuropharmacology. (2013) 69, 16–24, 10.1016/j.neuropharm.2012.04.004. 22521336 PMC3447120

[9] Furqan M. , Trofinetide-a New Chapter in Rett Syndrome’s Treatment, Frontiers in Pharmacology. (2023) 14, 10.3389/fphar.2023.1284035. PMC10687465 38035006

[10] Dolce A. , Ben-Zeev B. , Naidu S. , and Kossoff E. H. , Rett syndrome and Epilepsy: an Update for Child Neurologists, Pediatric Neurology. (2013) 48, no. 5, 337–345, 10.1016/j.pediatrneurol.2012.11.001. 23583050

[11] Operto F. F. , Mazza R. , Pastorino G. M. G. , Verrotti A. , and Coppola G. , Epilepsy and Genetics in Rett syndrome: A Review, Brain Behav. (2019) 9, no. 5, 10.1002/brb3.1250. PMC6520293 30929312

[12] Jian L. , Nagarajan L. , de Klerk N. , Ravine D. , Christodoulou J. , and Leonard H. , Seizures in Rett syndrome: An Overview from a One-Year Calendar Study, European Journal of Paediatric Neurology. (2007) 11, no. 5, 310–317, 10.1016/j.ejpn.2007.02.008. 17433737 PMC3013620

[13] Huppke P. , Köhler K. , Brockmann K. , Stettner G. M. , and Gärtner J. , Treatment of Epilepsy in Rett syndrome, European Journal of Paediatric Neurology. (2007) 11, no. 1, 10–16, 10.1016/j.ejpn.2006.09.003. 17178248

[14] Mukhtar I. , Inflammatory and Immune Mechanisms Underlying Epileptogenesis and Epilepsy: From Pathogenesis to Treatment Target, Seizure. (2020) 82, 65–79, 10.1016/j.seizure.2020.09.015. 33011590

[15] Meng F. and Yao L. , The Role of Inflammation in Epileptogenesis, Acta Epileptologica. (2020) 2, no. 1, 10.1186/s42494-020-00024-y.

[16] Palace J. and Lang B. , Epilepsy: an Autoimmune Disease?, Journal of Neurology Neurosurgery and Psychiatry. (2000) 69, no. 6, 711–714, 10.1136/jnnp.69.6.711. 11080217 PMC1737165

[17] Vezzani A. , Balosso S. , and Ravizza T. , Neuroinflammatory Pathways as Treatment Targets and Biomarkers in Epilepsy, Nature Reviews Neurology. (2019) 15, no. 8, 459–472, 10.1038/s41582-019-0217-x. 31263255

[18] Marchi N. , Granata T. , Ghosh C. , and Janigro D. , Blood-Brain Barrier Dysfunction and Epilepsy: Pathophysiologic Role and Therapeutic Approaches, Epilepsia. (2012) 53, no. 11, 1877–1886, 10.1111/j.1528-1167.2012.03637.x. 22905812 PMC4842020

[19] Bradshaw M. J. , Cho T. A. , and Chow F. C. , Central Nervous System Infections Associated with Immunosuppressive Therapy for Rheumatic Disease, Rheumatic Disease Clinics of North America. (2017) 43, no. 4, 607–619, 10.1016/j.rdc.2017.06.009. 29061246 PMC5679400

[20] Caporali R. , Cimmino M. A. , Ferraccioli G. et al., Systemic Vasculitis Study Group of the Italian Society for Rheumatology. Prednisone plus Methotrexate for Polymyalgia Rheumatica: A Randomized, Double-Blind, Placebo-Controlled Trial, Annals of Internal Medicine. (2004) 141, no. 7, 493–500, 10.7326/0003-4819-141-7-200410050-00005. 15466766

[21] Perpétuo I. P. , Caetano-Lopes J. , Rodrigues A. M. et al., Methotrexate and Low-Dose Prednisolone Downregulate Osteoclast Function by Decreasing Receptor Activator of Nuclear factor-κβ Expression in Monocytes from Patients with Early Rheumatoid Arthritis, RMD Open. (2017) 3, no. 1, 10.1136/rmdopen-2016-000365. PMC5604603 28955481

[22] Scholz J. , Abele A. , Marian C. et al., Low-Dose Methotrexate Reduces Peripheral Nerve Injury-Evoked Spinal Microglial Activation and Neuropathic Pain Behavior in Rats, Pain. (2008) 138, no. 1, 130–142, 10.1016/j.pain.2007.11.019. 18215468 PMC2536692

[23] Godhwani N. and Bahna S. L. , Minagar Alireza , Chapter 10-Immune Dysregulation in Epilepsy, Neuroinflammation. (2018) 2nd edition, Academic Press, 217–231, 10.1016/B978-0-12-811709-5.00011-9.

[24] Jefferson A. , Leonard H. , Siafarikas A. et al., Clinical Guidelines for the Management of Bone Health in Rett syndrome Based on Expert Consensus and Available Evidence, PLoS One. (2016) 11, no. 2, 10.1371/journal.pone.0146824. PMC4743907 26849438

[25] Hua J. , Huang J. , Li G. , Lin S. , and Cui L. , Glucocorticoid-Induced Bone Disorders in Children: Research Progress in Treatment Mechanisms, Frontiers in Endocrinology. (2023) 14, 10.3389/fendo.2023.1119427. PMC10111257 37082116

